# Safety, Pharmacokinetics, and Food Effect of Tebipenem Pivoxil Hydrobromide after Single and Multiple Ascending Oral Doses in Healthy Adult Subjects

**DOI:** 10.1128/AAC.00618-19

**Published:** 2019-08-23

**Authors:** Paul B. Eckburg, Akash Jain, Susannah Walpole, Grayson Moore, Luke Utley, Erika Manyak, Aaron Dane, David Melnick

**Affiliations:** a Spero Therapeutics, Cambridge, Massachusetts, USA; b DaneStat Consulting, Alderley Edge, United Kingdom

**Keywords:** carbapenem, oral, pharmacokinetics, tebipenem

## Abstract

Tebipenem pivoxil hydrobromide (TBPM-PI-HBr, formerly SPR994) is an orally available prodrug of tebipenem, a carbapenem with activity versus multidrug-resistant (MDR) Gram-negative pathogens, including quinolone-resistant and extended-spectrum-β-lactamase-producing Enterobacteriaceae. The safety and pharmacokinetics (PK) of tebipenem were studied after administration of single and multiple ascending oral doses of TBPM-PI-HBr in fed and fasted states. Healthy adults received single oral doses of TBPM-PI-HBr at 100 mg to 900 mg or placebo (*n* = 108) or multiple doses of 300 mg or 600 mg every 8 h or placebo (*n* = 16) for 14 days. In the single-ascending-dose (SAD) phase, mean tebipenem plasma concentrations increased in a linear and dose proportional manner for doses of 100 to 900 mg and were comparable in the fasted and fed states for the 300- and 600-mg doses. In the MAD phase, tebipenem maximum concentration (*C*_max_) was reached within 1.5 h and was dose proportional on day 1 and higher than dose proportional (2.7-fold) on day 14. AUC was more than 2-fold greater on day 1 (2.7-fold) and day 14 (2.5-fold) for 600 mg q8h than for 300 mg q8h. Approximately 55% to 60% of tebipenem was recovered in the urine. TBPM-PI-HBr was well tolerated; mild, transient diarrhea was the most commonly reported adverse event. TBPM-PI-HBr provides an orally bioavailable carbapenem option to treat serious infections caused by MDR Enterobacteriaceae and has the potential to decrease the need for intravenous antibiotic therapy in the hospital or outpatient setting. (This study has been registered at ClinicalTrials.gov under identifier NCT03395249.)

## Abstract (precis)

Tebipenem pivoxil hydrobromide (TBPM-PI-HBr, formerly SPR994) is an orally available prodrug of tebipenem, a carbapenem with activity versus multidrug-resistant (MDR) Gram-negative pathogens, including quinolone-resistant and extended-spectrum-β-lactamase-producing Enterobacteriaceae. The safety and pharmacokinetics (PK) of tebipenem were studied after administration of single and multiple ascending oral doses of TBPM-PI-HBr in fed and fasted states.

## INTRODUCTION

In recent years, an increased rate of infections caused by multidrug-resistant (MDR) Gram-negative pathogens has been recognized as a serious threat and public health concern (1, 2). For patients with serious infections due to MDR pathogens, limited treatment options represent a challenge to effective management (3–5).

Tebipenem pivoxil hydrobromide (TBPM-PI-HBr; formerly SPR994) is the oral prodrug of tebipenem under development as an alternative to intravenous (i.v.) carbapenem antibiotic therapy. TBPM-PI-HBr is rapidly converted to active tebipenem in plasma and enterocytes. Tebipenem is a carbapenem with activity against multidrug-resistant Gram-negative pathogens, including quinolone-resistant and extended-spectrum-β-lactamase (ESBL)-producing Enterobacteriaceae. Tebipenem demonstrates potent *in vitro* microbiological activity against a wide variety of Gram-negative pathogens, including MDR strains (6–10), and *in vivo* efficacy in murine models of soft tissue, pulmonary, and urinary tract infections (11–13). Results from *in vitro* and *in vivo* infection models indicate that time-dependent pharmacokinetic/pharmacodynamic (PK/PD) parameters (cumulative percentage [expressed as a percentage of the dosing interval] or time [in hours], respectively, of a 24-h period that the drug concentration exceeds the MIC under steady-state pharmacokinetic conditions [%*fT*_>MIC_] and free drug area under the curve [*f*AUC]/MIC·1/tau, where tau represents the length of the dosing interval) are most predictive of antimicrobial activity of tebipenem (14, 15). In a hollow-fiber model, the %*fT*_>MIC_ ranged from 28% to 100% (14).

We report results from a study assessing the safety, PK, and food effect of TBPM-PI-HBr after a single ascending dose (SAD) and multiple ascending oral doses (MAD) in fed and fasted states in healthy subjects.

## RESULTS

### Subject disposition and baseline characteristics.

In the SAD phase, 108 subjects were randomized and analyzed for safety, and 75 in the TBPM-PI-HBr group and 8 in the Orapenem group provided PK data. In the MAD phase, 16 subjects were randomized and analyzed for safety, and 12 subjects who received tebipenem provided PK data. In the SAD phase, 2 subjects were withdrawn from the study because they were lost to follow-up (1 subject) or withdrew consent (1 subject). Two additional subjects were withdrawn from dosing due to treatment-emergent adverse events (TEAEs) of serum alanine aminotransferase (ALT) increases (1 Orapenem, and 1 placebo) but continued study visits. Across treatment groups, subjects in the SAD and MAD phases were comparable for baseline demographics (Table 1).

**TABLE 1:** Baseline demographics for subjects in the SAD and MAD phases

Parameter	Single-ascending-dose phase			Multiple-ascending-dose phase	
	Total TBPM-PI-HBr (*n* = 75)	Placebo (*n* = 25)	Orapenem (*n* = 8)	Total TBPM-PI-HBr (*n* = 12)	Placebo (*n* = 4)
Age, yrs^*a*^	26.9 ± 6.7	27.0 ± 8.7	25.6 ± 2.4	24.8 ± 4.5	28.5 ± 4.7
Male, no. (%)	75 (100)	25 (100)	8 (100)	12 (100)	4 (100)
Race, no. (%)					
White	56 (75)	18 (72)	7 (88)	8 (67)	3 (75)
Asian	15 (20)	7 (28)	1 (13)	4 (33)	0
Black or African American				0	1 (25)
Other	4 (5)	0	0		
Wt (kg)^*a*^	75.4 ± 10.1	79.6 ± 8.9	71.6 ± 5.5	74.4 ± 7.0	74.1 ± 4.0
Ht (cm)^*a*^	177.7 ± 6.9	178.5 ± 5.9	176.9 ± 8.0	177.2 ± 6.7	176.6 ± 3.3
Body mass index (kg/m^2^)^*a*^	23.9 ± 3.1	25.0 ± 2.7	22.9 ± 1.5	23.7 ± 2.1	23.8 ± 1.9

a Mean ± SD.

### Pharmacokinetics.

**FIG 1:**
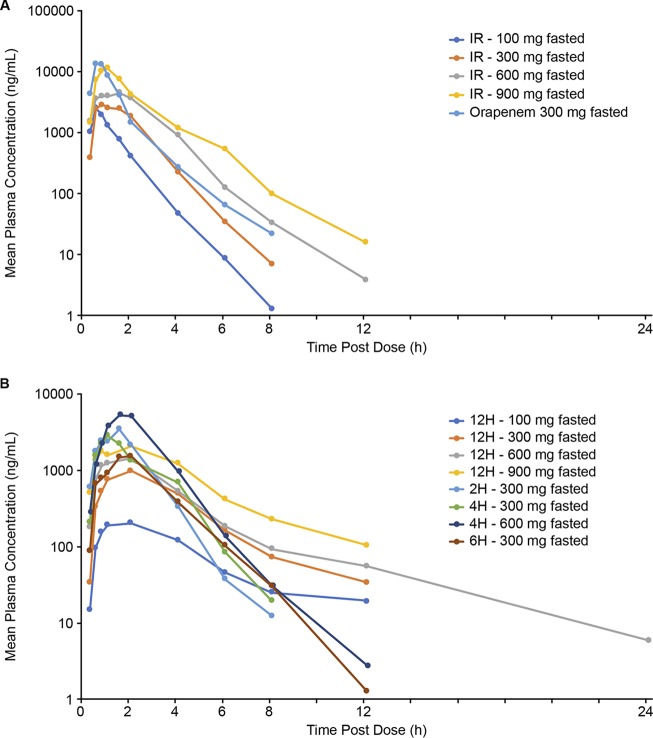
Mean plasma tebipenem concentrations (semilog) during fasting in the SAD phase for IR formulations (A) and ER formulations (B).

**(i) SAD phase.** Both immediate-release (IR) and extended-release (ER) formulations of TBPM-PI-HBr were evaluated in the single-dose phase of this study. Following fasted administration of IR formulations of TBPM-PI-HBr, plasma exposure (maximum concentration in serum [*C*_max_] and AUC) increased with dose over the range from 100 mg to 900 mg. Median time to maximum concentration in serum (*T*_max_) ranged from 0.5 to 1.3 h and mean half-life from 0.8 to 1.1 h (Table 2 and Fig. 1). In comparison, tebipenem exposure (*C*_max_ and AUC_last_) following fasted dose administration of TBPM-PI-HBr was lower for the ER 12-h and 6-h formulations than for the IR and 2-h and 4-h ER formulations (Table 2 and Fig. 1).

**TABLE 2:** Plasma PK parameters for tebipenem during the fasted state in SAD phase (PK analysis population)

Drug and dose	Median (range) for *T*_max_ (h)	Arithmetic mean (% CV)					
		*C*_max_ (ng/ml)	AUC_last_ (h·ng/ml)	AUC_0–∞_ (h·ng/ml)	*t*_1/2_ (h)	Nominal dose	
						CL (liters/h)	*V* (liters)
TBPM-PI-HBr							
12 h, 100 mg (*n* = 6)	1.5 (0.75–4.0)	256 (37.3)	923 (45.4)	872^*a*^ (15.2)	2.0^*a*^ (26.9)	89.9^*a*^ (16.7)	267.2^*a*^ (37.7)
12 h, 300 mg (*n* = 6)	2.0 (1.02–4.0)	1,209 (38.2)	3,738 (26.1)	—^*b*^	—^*b*^	—^*b*^	—^*b*^
12 h, 600 mg (*n* = 9)	1.0 (0.5–4.0)	1,944 (40.6)	5,502 (34.7)	5,192^*c*^ (29.9)	3.8^*c*^ (120.3)	95.2^*c*^ (25.4)	428.3^*c*^ (79.0)
12 h, 900 mg (*n* = 6)	1.5 (0.75–4.0)	2,943 (35.6)	9,180 (34.6)	10,571^*d*^ (20.1)	2.5^*d*^ (28.6)	68.2^*d*^ (24.0)	242.3^*d*^ (37.0)
2 h, 300 mg (*n* = 6)	1.5 (0.5–2.0)	4,062 (13.3)	7,253 (12.9)	7,268 (12.9)	0.8 (7.3)	32.3 (15.0)	38.7 (14.2)
4 h, 300 mg (*n* = 6)	1.0 (0.5–4.0)	3,064 (16.1)	6,450 (18.6)	6,267^*d*^ (19.4)	0.8^*d*^ (13.2)	38.0^*d*^ (18.6)	45.8^*d*^ (23.3)
4 h, 600 mg (*n* = 6)	1.75 (1.0–2.0)	6,216 (33.2)	13,577 (19.8)	13,602 (19.7)	1.0 (30.6)	35.2 (21.8)	53.1 (35.8)
6 h, 300 mg (*n* = 6)	1.5 (0.5–2.0)	1,810 (24.4)	4,410 (26.4)	4,456 (25.4)	1.2 (27.2)	54.7 (25.0)	94.8 (41.4)
IR, 100 mg (*n* = 6)	0.5 (0.25–0.85)	2,893 (38.9)	2,846 (31.7)	2,875 (30.7)	0.9 (48.7)	29.3 (33.8)	34.3 (45.6)
IR, 300 mg (*n* = 6)	1.1 (0.5–2.0)	4,006 (41.9)	6,473 (29.7)	6,488 (29.6)	0.8 (21.0)	39.1 (36.3)	46.2 (49.6)
IR, 600 mg (*n* = 6)	1.3 (0.5–2.0)	6,203 (31.7)	12,693 (30.4)	12,715 (30.3)	1.1 (24.5)	39.4 (31.4)	61.4 (47.5)
IR, 900 mg (*n* = 6)	1.0 (0.75–1.5)	12,652 (47.9)	21,862 (23.9)	21,913 (23.9)	1.0 (25.6)	33.5 (28.1)	47.2 (31.5)
Orapenem, 300 mg (*n* = 8)	0.5 (0.5–0.75)	15,737 (23.3)	15,569 (30.7)	15,601 (30.6)	1.0 (14.9)	21.1 (35.2)	31.3 (50.7)

a *n* = 3.

b —, *n* = 2.

c *n* = 8.

d *n* = 5.

**FIG 2:**
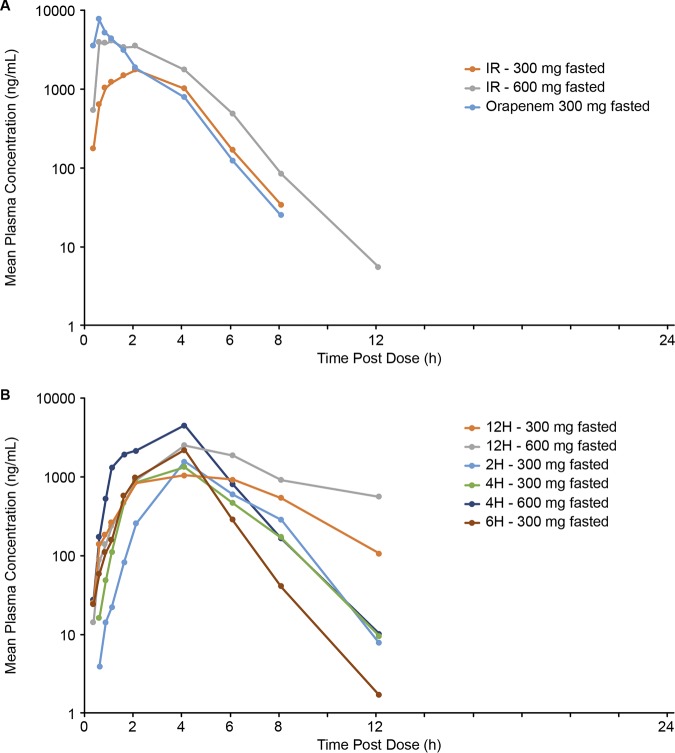
Mean plasma tebipenem concentrations (semilog) during the fed state in the SAD phase for IR formulations (A) and ER formulations (B).

Administration of TBPM-PI-HBr following a standard high-fat meal had a variable effect on plasma exposure for the different tablet formulations. Following the fed dose administration of TBPM-PI-HBr, a reduction of approximately 50% in mean *C*_max_ was observed for the 300-mg IR dose, but the mean AUC exposures at this dose were similar following fasted and fed administrations. Furthermore, plasma exposures (both *C*_max_ and AUC) were similar following fasted and fed administrations of TBPM-PI-HBr at 600 mg (IR) (Table 3 and Fig. 2). For the 6-h and 12-h ER formulations of TBPM-PI-HBr at 300 mg, an increase in AUC and *C*_max_ was observed when administered in the fed versus fasted state. This was not observed following fed administration of the 2-h and 4-h ER formulations (Table 3 and Fig. 2). There was generally a linear relationship between dose and exposure following administration in the fasted (Fig. 1) and fed (Fig. 2) states.

**TABLE 3:** Plasma PK parameters for tebipenem during the fed state in SAD phase (PK analysis population)

Drug and dose	Median (range) for *T*_max_ (h)	Arithmetic mean (% CV)					
		*C*_max_ (ng/ml)	AUC_last_ (h·ng/ml)	AUC_0–∞_ (h·ng/ml)	*t*_1/2_ (h)	Nominal dose	
						CL (liters/h)	*V* (liters)
TBPM-PI-HBr							
12 h, 300 mg (*n* = 6)	5.0 (2.0–8.0)	1,892 (51.0)	7,175 (38.5)	—^*a*^	—^*a*^	—^*a*^	—^*a*^
12 h, 600 mg (*n* = 6)	4.0 (4.0–12.0)	3,014 (37.5)	14,213 (32.0)	14,727^*b*^ (37.0)	1.3^*b*^ (25.9)	35.3^*b*^ (44.9)	70.6^*b*^ (71.6)
2 h, 300 mg (*n* = 6)	4.0 (4.0–8.0)	1,852 (37.7)	5,528 (23.8)	6,215^*b*^ (14.8)	1.1^*b*^ (5.3)	37.8^*b*^ (16.1)	59.1^*b*^ (11.3)
4 h, 300 mg (*n* = 6)	4.0 (2.0–6.0)	1,677 (50.5)	5,417 (31.2)	6,549^*b*^ (0.2)	0.9^*b*^ (17.5)	35.3^*b*^ (0.2)	43.9^*b*^ (17.7)
4 h, 600 mg (*n* = 6)	4.0 (1.5–6.0)	5,830 (56.4)	15,363 (39.3)	16,547^*c*^ (39.9)	1.1^*c*^ (31.3)	31.9^*c*^ (43.4)	53.6^*c*^ (74.4)
6 h, 300 mg (*n* = 6)	4.0 (2.0–4.0)	2,288 (32.7)	6,579 (16.0)	—^*d*^	—^*d*^	—^*d*^	—^*d*^
IR, 300 mg (*n* = 6)	2.0 (0.5–4.0)	2,058 (31.8)	6,169 (21.3)	6,137^*e*^ (23.7)	0.9^*e*^ (9.3)	39.5^*e*^ (24.5)	49.0^*e*^ (32.6)
IR, 600 mg (*n* = 6)	1.5 (0.5–4.0)	6,451 (73.7)	14,160 (42.4)	14,200 (42.4)	0.9 (15.8)	37.7 (43.2)	44.9 (34.1)
Orapenem, 300 mg (*n* = 7)	0.5 (0.5–1.0)	8,718 (40.3)	11,321 (29.6)	11,352 (29.7)	0.8 (17.1)	28.7 (32.3)	32.6 (26.4)

a —, *n* = 0.

b *n* = 3.

c *n* = 4.

d —, *n* = 2.

e *n* = 5.

The PK profile of Orapenem at 300 mg during fasting was characterized by a *T*_max_ of 0.5 h and half-life of 1.0 h, with *C*_max_ exceeding that of the TBPM-PI-HBr IR formulations (Table 2 and Fig. 1). With Orapenem, a reduction in both AUC and *C*_max_ but no change in *T*_max_ was observed when administered in fed compared with fasted states (Table 3 and Fig. 2).

**FIG 3:**
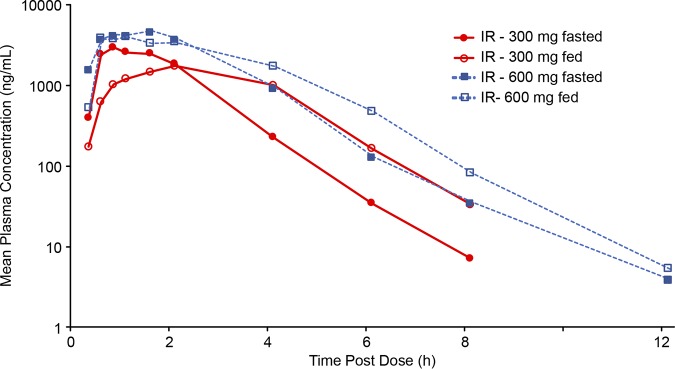
Mean plasma concentrations (semilog) of tebipenem (300- and 600-mg IR formulations) during the SAD phase, fasted versus fed.

A summary of the effect of food on the relative bioavailability of tebipenem following administration of the IR and ER formulations of TBPM-PI-HBr and of Orapenem is presented in Table 4. Given that the 6-h and 12-h ER formulations were associated with decreased absorption, the fact that the relative bioavailability associated with the 2-h and 4-h ER formulations was not substantially better than that of the IR formulation, and the predictability of IR formulation PK characteristics over the range of doses studied, the IR formulation was utilized in the MAD phase of the study. Of note, food did not impact the AUC exposure observed following administration of 300 mg (IR) or 600 mg (IR) of TBPM-PI-HBr (Fig. 3).

**TABLE 4:** Summary of food effect on relative bioavailability of tebipenem in the SAD phase

Drug and dose	AUC_last_		*C*_max_	
	% ratio of LS^*a*^ means (fasted/fed)	90% CI	% ratio of LS means (fasted/fed)	90% CI
TBPM-PI-HBr				
12 h, 300 mg (*n* = 6)	185.9	146.9, 235.1	148.6	107.3, 205.8
12 h, 600 mg (*n* = 6)	273.5	194.8, 384.1	166.2	101.2, 272.7
2 h, 300 mg (*n* = 6)	74.9	58.4, 96.0	43.3	30.7, 61.2
4 h, 300 mg (*n* = 6)	81.1	63.0, 104.3	47.6	25.6, 88.5
4 h, 600 mg (*n* = 6)	107.7	73.6, 157.5	83.6	46.1, 151.5
6 h, 300 mg (*n* = 6)	151.9	125.3, 184.3	124.3	96.4, 160.3
IR, 300 mg (*n* = 6)	97.5	71.1, 133.8	52.9	31.7, 88.3
IR, 600 mg (*n* = 6)	108.1	73.9, 158.0	90.3	41.7, 195.6
Orapenem, 300 mg (*n* = 7)	70.2	61.6, 80.0	50.1	32.2, 77.8

a LS, least square.

**FIG 4:**
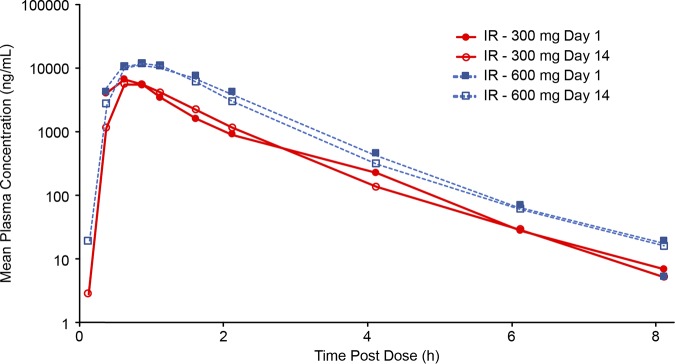
Mean plasma tebipenem concentrations (semilog) in MAD phase at day 1 and day 14.

**(ii) MAD phase.** Only the IR formulation of TBPM-PI-HBr (300 mg and 600 mg) was evaluated in the MAD phase of this study. *C*_max_ was reached within 1.5 h of dose administration on both day 1 (single dose) and day 14 (steady state), with a median *T*_max_ of less than 1 h (Table 5). Pharmacokinetic parameters of exposure increased more rapidly than dose, with AUC for the 600-mg dose being more than twice the AUC for the 300-mg dose on both day 1 (2.7-fold) and day 14 (2.5-fold). *C*_max_ was dose proportional on day 1 and higher than dose proportional (2.7-fold) on day 14 for the 600-mg than for the 300-mg dose (Fig. 4). No accumulation occurred at a tebipenem doses of 300 mg and 600 mg every 8 h (q8h). The accumulation ratio of AUC from 0 to 8 h (AUC_0–8_) for day 14 versus day 1 was 1.01 for the 300-mg dose and 0.87 for the 600-mg dose, which was consistent with a short half-life (<1 h) for tebipenem.

**TABLE 5:** Plasma PK parameters in oral TBPM-PI-HBr given q8h for 14 days in the MAD phase (PK analysis population)

Day	TBPM-PI-HBr dose	Median (range) for *T*_max_ (h)	Arithmetic mean (% CV)				
			*C*_max_ (ng/ml)	AUC_0–8_ (h·ng/ml)	*t*_1/2_ (h)	Nominal dose	
						CL (liters/h)	*V* (liters)
1	300 mg (*n* = 6)	0.5 (0.25–1.0)	7,759 (50.7)	7,726 (27.2)	0.82 (26.9)	32.4 (35.6)	37.0 (28.2)
	600 mg (*n* = 6)	0.88 (0.5–1.5)	13,428 (31.9)	20,592 (19.3)	0.79 (12.1)	23.2 (19.5)	26.2 (19.2)
14	300 mg (*n* = 6)	0.63 (0.47–1.5)	6,493 (61.5)	7,484 (36.5)	0.72 16.0)	34.8 (39.3)	36.5 (47.8)
	600 mg (*n* = 6)	0.63 (0.5–1.5)	15,090 (30.8)	17,924 (25.4)	0.83 (20.0)	27.5 (30.1)	31.8 (21.4)

### Urine concentrations.

In the SAD phase, the mean fraction of the administered dose excreted in urine as unchanged drug (tebipenem) with fasted administration of the IR and 2-h, 4-h, and 6-h ER formulations of TBPM-PI-HBr ranged from 35.0% to 59.2% and during fed administration from 45.1% to 61.8% (Table 6); the fractions excreted in urine were similar for the IR formulation and for Orapenem (59.2% fasted and 55.1% fed). For the 12-h ER formulation of TBPM-PI-HBr, the mean fractions of dose excreted in urine were 20.8% to 28.5% with fasted administration and 53.8% to 62.7% with fed administration. Renal clearance ranged from 12.8 to 22.7 liters/h and was not affected by food. In the MAD phase, 57% and 66% of tebipenem for the 300-mg and 600-mg doses of TBPM-PI-HBr were excreted in urine on day 1. The fraction excreted (Fe; 0 to 8 h) was lower on day 14 (39.4% and 28.8%, respectively). Renal clearances were 15.2 to 16.7 liters/h on day 1 and 7.5 to 11.9 liters/h on day 14.

**TABLE 6:** Excretion of TBPM-PI-HBr in urine and renal clearance for SAD and MAD phases

Phase, drug, and dose	Mean (% CV)					
	Fasted (day 1)			Fed (day 7)		
	Fe, 0–4 h	Fe, 0–24 h	CL_R_,^*d*^ 0–24 h (liters/h)	Fe, 0–4 h	Fe, 0–24 h	CL_R_, 0–24 h (liters/h)
SAD phase						
TBPM-PI-HBr						
12 h, 100 mg (*n* = 6)	13.8 (40.2)	21.4 (40.4)	18.5 (21.0)			
12 h, 300 mg (*n* = 6)	19.8 (34.9)	28.5 (31.4)	17.6 (20.9)	17.0 (99.0)	53.8 (14.1)	19.0 (29.0)
12 h, 600 mg (*n* = 9)	14.4 (36.0)	20.8 (28.6)	18.9 (45.5)	13.8^*a*^ (97.0)	62.7^*a*^ (22.7)	21.8^*a*^ (28.6)
12 h, 900 mg (*n* = 6)	15.0 (34.2)	24.6 (22.5)	19.7 (22.0)			
2 h, 300 mg (*n* = 6)	55.7 (16.1)	59.1 (14.1)	18.9 (11.9)	13.2 (119.4)	45.1 (39.8)	19.9 (50.0)
4 h, 300 mg (*n* = 6)	35.8 (32.9)	40.3 (19.3)	15.2 (34.7)	25.6 (80.7)	50.9 (29.3)	22.7 (38.5)
4 h, 600 mg (*n* = 6)	49.9 (27.3)	54.9 (25.8)	18.6 (14.4)	31.3 (86.5)	61.8 (30.9)	20.1 (41.0)
6 h, 300 mg (*n* = 6)	29.1 (14.8)	35.0 (13.7)	19.0 (19.0)	32.8 (34.8)	58.0 (12.0)	20.9 (21.7)
IR, 100 mg (*n* = 6)	58.4 (25.9)	59.7 (25.7)	17.0 (28.5)			
IR, 300 mg (*n* = 6)	44.6 (23.4)	47.1 (22.1)	17.5 (19.1)	34.9 (41.0)	46.0 (18.5)	17.5 (16.8)
IR, 600 mg (*n* = 6)	44.5 (22.0)	47.8 (20.4)	18.2 (22.7)	37.6 (63.0)	49.2 (37.5)	16.4 (22.2)
IR, 900 mg (*n* = 6)	46.2 (47.0)	53.5 (27.6)	17.0 (18.5)			
Orapenem 300 mg (*n* = 8)	58.3 (13.1)	59.2 (13.2)	12.8 (43.0)	50.9^*b*^ (20.8)	55.1^*b*^ (22.3)	15.7^*b*^ (33.4)
MAD phase						
Orapenem						
300 mg q8h (*n* = 6)	56.0 (35.2)	56.9 (34.6)	16.7 (26.0)	35.7 (76.5)	39.4^*c*^ (63.7)	11.9^*c*^ (56.8)
600 mg q8h (*n* = 6)	63.7 (9.6)	65.6 (7.9)	15.2 (20.3)	27.4 (94.7)	28.8 (88.4)	7.5 (89.3)

a *n* = 6.

b *n* = 7.

c *n* = 4.

d CL_R_, renal clearance.

### Safety and tolerability.

A total of 58 TEAEs were reported for 35 of 108 subjects (32%) in the SAD phase, including 20 of 75 (27%) TBPM-PI-HBr-treated subjects, 5 of 8 (63%) Orapenem-treated subjects, and 10 of 25 (40%) placebo-treated subjects (Table 7). Most TEAEs (55 of 58 [95%]) were mild in severity. Two TEAEs were of moderate severity {1 TEAE of unrelated conjunctivitis in the TBPM-PI-HBr (600 mg) 12-h cohort and 1 TEAE of probably-related ALT increase (>3× to 5× the upper limit of normal [ULN]) in the Orapenem cohort}. One TEAE was severe (probably-related ALT increase [>5× to 10× ULN] in the placebo group). The last 2 subjects with TEAEs of ALT increased (1 Orapenem-treated subject and 1 placebo-treated subject) were the only subjects in the SAD phase with premature discontinuation of study drug, both due to elevated serum aminotransferases (elevated ALT and aspartate aminotransferase [AST]). No TBPM-PI-HBr-treated subject had premature discontinuation of study drug or withdrawal from the study. No subject in the SAD phase experienced a serious AE or death.

**TABLE 7:** Incidence of AEs occurring with TBPM-PI-HBr in SAD and MAD phases

AE(s)	No. (%) of subjects [no. of events]					
	SAD phase			MAD phase		
	TBPM-PI-HBr	Orapenem	Placebo	TBPM-PI-HBr		Placebo
	All SAD (*n* = 75)	300 mg (*n* = 8)	(*n* = 25)	300 mg (*n* = 6)	600 mg (*n* = 6)	(*n* = 4)
Treatment-emergent AEs	20 (27) [33]	5 (63) [10]	10 (40) [15]	6 (100) [12]	6 (100) [16]	4 (100) [6]
Treatment-related AEs	11 (15) [14]	3 (38) [5]	3 (12) [5]	6 (100) [9]	5 (83) [11]	2 (50) [4]
Abdominal discomfort	0	0	1 (4) [1]	1 (17) [1]	0	0
Abdominal distention	0	0	0	0	0	1 (25) [1]
Abdominal pain	0	0	0	1 (17) [1]	1 (17) [1]	1 (25) [1]
Abdominal pain, upper	0	0	0	0	2 (33) [2]	1 (25) [1]
Alanine aminotransferase increase	0	1 (13) [1]	1 (4) [1]	2 (33) [2]	1 (17) [1]	0
Aspartate aminotransferase increase	0	1 (13) [1]	1 (4) [1]	1 (17) [1]	0	0
Diarrhea	6 (8) [6]	0	1 (4) [1]	2 (33) [2]	5 (83) [5]	1 (25) [1]
Dizziness	1 (1) [1]	0	0	0	0	0
Dry mouth	1 (1) [1]	0	0	1 (17) [1]	0	0
Gamma glutamyl transferase increase	0	0	1 (4) [1]	0	1 (17) [1]	1 (25) [1]
Abnormal gastrointestinal sounds	1 (1) [1]	0	0	0	0	0
Headache	3 (4) [5]	2 (25) [3]	0	1 (17) [1]	1 (17) [1]	0

Among all TBPM-PI-HBr-treated subjects, there were no commonly occurring TEAEs, i.e., TEAEs reported for at least 8 (≥10%) TBPM-PI-HBr-treated subjects. The most common TEAEs in the total TBPM-PI-HBr group were diarrhea (6 [8%] subjects, 1 subject in each of the 100-mg IR, 300-mg 4-h, 600-mg 6-h, 100-mg 12-h, 600-mg 12-h, and 900-mg 12-h cohorts) and headache (4 [5%] subjects, 1 subject in each of the 100-mg IR, 300-mg 2-h, 600-mg 4-h, and 300-mg 6-h cohorts). No other individual TEAEs were reported for more than 3 subjects.

Events of diarrhea were assessed in detail (6 TBPM-PI-HBr-treated subjects and 1 placebo-treated subject). There was no trend in diarrhea TEAEs with respect to TBPM-PI-HBr dosage or fed versus fasting status. The 6 diarrhea TEAEs in the TBPM-PI-HBr-treated subjects occurred in 6 different dosage cohorts (ranging from 100 mg to 900 mg), and the events occurred under either the fed (3/7 events) or fasting (4/7 events) condition. All events of diarrhea were deemed mild in severity by the principal investigator. The time to onset of all diarrhea TEAEs was <1 day from dosing, with the exception of an unknown time to onset in the 100-mg 12-h subject, and most events resolved within 1 day. There were no cases of Clostridium difficile infection. In addition, gastrointestinal events of nausea and vomiting were not observed in any subject in the SAD phase, and no TBPM-PI-HBr-treated subject had ALT or AST elevation over 3× the ULN.

In the MAD phase, a total of 34 TEAEs were reported for 16 of 16 (100%) subjects (12 TBPM-PI-HBr-treated subjects and 4 placebo-treated subjects). All TEAEs were mild in severity, except for 1 moderately severe TEAE (1 event of ALT increased [>3× to 5× the ULN] in a subject treated with TBPM-PI-HBr at 300 mg q8h). No subject in the MAD phase experienced a severe TEAE, TEAE that led to premature discontinuation of study drug or study withdrawal, or a serious AE. The moderate ALT increase in the subject treated with TBPM-PI-HBr at 300 mg q8h was deemed probably related to study treatment. This TEAE commenced 6 days following first dose administration and resolved 12 days later. This subject had 2 doses temporarily held (dose 3 on day 8 and dose 1 on day 9), due to the AEs of ALT and AST increases. Follow-up unscheduled serum aminotransferase values on day 8 and day 9 were trending down from peak levels; therefore, a decision was made to restart TBPM-PI-HBr. The subject completed the remainder of TBPM-PI-HBr doses, with an overall treatment compliance of 95% (38/40 doses). Of note, the aminotransferase levels did not worsen after rechallenge with TBPM-PI-HBr.

The most common type of TEAE by system organ class in both treatment groups was gastrointestinal disorders (11 [69%] TBPM-PI-HBr-treated subjects and 2 [50%] placebo-treated subjects), consisting primarily of diarrhea (2 subjects treated with TBPM-PI-HBr at 300 mg q8h, 5 subjects treated with TBPM-PI-HBr at 600 mg q8h, and 1 placebo-treated subject). Other commonly occurring TEAEs in the TBPM-PI-HBr cohorts, i.e., TEAEs reported for at least 2 (≥10%) of TBPM-PI-HBr-treated subjects, included headache (2 subjects), abdominal pain (2 subjects), and ALT increase (3 subjects).

Adverse events determined to be possibly or probably related to study drug were reported for 13 (92%) TBPM-PI-HBr-treated subjects and 4 (100%) placebo-treated subjects. All study drug-related AEs in the MAD phase were of mild severity, except for the single case of moderate ALT increase in the subject treated with TBPM-PI-HBr at 300 mg q8h described above. All gastrointestinal TEAEs (9 TBPM-PI-HBr-treated subjects and 2 placebo-treated subjects) and aminotransferase elevation TEAEs (3 TBPM-PI-HBr-treated subjects) in the MAD phase were deemed possibly or probably related to study drug.

Although limited by the small numbers of subjects per cohort (6 subjects per TBPM-PI-HBr cohort), more TEAEs of diarrhea occurred in the higher-dose cohort (600 mg q8h) than in the 300-mg q8h group or placebo group. However, all events of diarrhea were deemed mild in severity by the principal investigator. The time to onset of all diarrhea TEAEs was <1 day from dosing, and most events resolved within 1 day (2 of the 8 diarrhea TEAEs resolved in approximately 2 days). There were no cases of C. difficile infection. Vomiting was not observed in any subject in the MAD phase; however, 1 subject treated with TBPM-PI-HBr at 600 mg q8h and 1 placebo-treated subject experienced nausea (both cases mild and possibly related to study drug). Finally, with the exception of the single moderate TEAE of ALT increase described above, no subject in the MAD phase had ALT elevations >3× the ULN.

No clinically significant findings were observed for physical examinations, vital signs, clinical laboratory testing, or electrocardiogram (ECG) testing in either the SAD or MAD phase.

## DISCUSSION

TBPM-PI-HBr is being developed as an oral carbapenem for the treatment of serious infections caused by MDR Gram-negative pathogens, with the potential opportunity for avoidance of hospitalization and/or to transition patients home more quickly after initiating therapy with i.v. antibiotics in the hospital. Unlike other carbapenems used to treat MDR infections in adults, TBPM-PI-HBr is an orally administered tablet formulation that provides high tebipenem bioavailability (50% to 60%). Thus, oral administration may allow physicians to avoid or limit the duration of i.v. antibiotics, provide an oral carbapenem option as step-down therapy from i.v. carbapenem therapy, or allow for a reduction in costs associated with avoiding hospitalization.

Results from this study demonstrate that the PK profile of tebipenem generally was dose proportional and linear after single doses of 100 to 900 mg with the IR formulation. Results from the MAD phase indicate dose proportionality and approximately linear PK with 300 and 600 mg q8h, with no accumulation over 14 days. While *C*_max_ was lower with the 300-mg dose of the IR formulation during the fed state, exposure (AUC from 0 h to infinity [AUC_0-∞_]) was proportional for both the 300-mg IR and 600-mg IR doses between fed and fasted states, supporting administration of TBPM-PI-HBr without respect to meals. More variability in the PK profile of the ER formulations was observed across doses. While this study examined the various ER formulations to determine PK properties and their potential for extending the dosage interval, based on results during the SAD phase, the IR formulation will be used in future studies. Studies of PK in patients with serious infections are needed to confirm these results.

In this SAD/MAD study, TBPM-PI-HBr was well tolerated. Gastrointestinal events were the most common types of TEAEs in both the SAD and MAD parts of the study (whether in TBPM-PI-HBr-treated subjects or control groups), predominantly consisting of transient, mild events of loose stools that occurred on the first day of dosing and resolved spontaneously within 24 h. In the MAD study, these events resolved within 1 to 2 days despite ongoing study drug dosing q8h for the full 14-day course. There were no cases of Clostridium difficile infection. Gastrointestinal events such as diarrhea are common, well-described effects of the β-lactam antibiotic class.

Of note, plasma concentrations of tebipenem at day 1 with the 300- and 600-mg doses of TBPM-PI-HBr exceeded the MIC_90_ for Klebsiella pneumoniae (0.06 ng/ml) and Escherichia coli (0.03 ng/ml) for 4 h, which is 50% of the q8h dosing interval. Urine concentrations of tebipenem were 50- to 100-fold greater than free plasma tebipenem concentrations. Thus, urine concentrations exceeded the MIC_90_ of 0.03 ng/ml for 24 h with single oral doses of 300 or 600 mg of TBPM-PI-HBr. Consequently, TBPM-PI-HBr should prove valuable as an oral agent for treating patients with complicated urinary tract infection and acute pyelonephritis.

Carbapenems have emerged as the standard of care for multiple types of MDR Gram-negative bacterial infections, but carbapenems currently are available only as i.v. formulations, highlighting the unmet need for an oral formulation of carbapenems to treat serious infections due to MDR pathogens. Results from *in vitro* studies demonstrated that tebipenem has potent antibacterial activity against MDR strains, including ESBL-producing Enterobacteriaceae (6–8). Combined with the promising PK and tolerability of its orally available TBPM-PI-HBr formulation, tebipenem is well positioned to address this unmet need.

An unmet medical need exists for safe and effective oral treatment options directed against serious infections caused by MDR Gram-negative pathogens, such as ESBL-producing or quinolone-resistant Enterobacteriaceae. The data described here provide evidence in support of the safe administration of TBPM-PI-HBr orally q8h for up to 14 days in healthy adults. Thus, oral TBPM-PI-HBr dosed at 600 mg q8h provides a highly bioavailable oral carbapenem to support the treatment of serious infections caused by cephalosporin- and fluoroquinolone-resistant Enterobacteriaceae, such as complicated urinary tract infection and acute pyelonephritis, and has the potential to decrease the need for i.v. antibiotic therapy in the hospital or outpatient setting.

## MATERIALS AND METHODS

This study was conducted according to the principles of the Declaration of Helsinki and Guidance on Good Clinical Practice. The study protocol, amendments, and informed consent forms were reviewed and approved by an Institutional Review Board. All subjects provided written informed consent prior to participating in any study activities. This study was registered at Clinicaltrials.gov under registration number NCT03395249.

### Investigational products.

For this study, TBPM-PI-HBr was formulated as IR and ER oral tablets containing TBPM-PI at 100, 300, or 600 mg. Multiple TBPM-PI-HBr formulations of various release times, including IR, 2-h release, 4-h release, 6-h release, and 12-h release, were tested. Placebo tablets (100, 300, and 600 mg) were pressed from a single placebo blend consisting of the same inactive ingredients as TBPM-PI-HBr; the active pharmaceutical ingredient was replaced by mannitol 200SD. Orapenem fine granules (containing 65 mg of TBPM-PI, equivalent to 50 mg of TBPM per sachet) were manufactured by Meiji Seika Pharma Co., Tokyo, Japan.

### Study design.

This was a double-blind, placebo-controlled, ascending-dose, multicohort study. The study was conducted in two parts: a SAD phase, followed by a MAD phase. Each phase of the study consisted of a screening period, a treatment period, and a follow-up period. The sponsor, the principal investigator, clinical study personnel participating in the study, and subjects were blinded to treatment assignment.

In sequential SAD cohorts, 8 subjects were randomized per cohort in a 3:1 ratio to receive TBPM-PI-HBr at 100, 300, 600, or 900 mg in various IR and ER tablet formulations or placebo (TBPM-PI-HBr dosages indicate amounts of TBPM-PI; each 300-mg dose of TBPM-PI contains 231 mg of active TBPM) (Table 8). Subjects in cohorts 1 and 7 to 14 received a single dose of TBPM-PI-HBr or placebo in a fasted state (at least a 10-h fast) and a second dose after a 5-day washout following a standardized high-fat meal (approximately 930 kcal) to investigate the food effect on the PK of tebipenem (Table 8). Subjects in cohorts 3, 6, 16, and 17 received a single dose of TBPM-PI-HBr in the fasted state only. Subjects in cohort 2 (TBPM-PI-HBr at 600 mg in a 12-h ER formulation) initially received a single dose of TBPM-PI-HBr or placebo in a fasted state, and 5 of these subjects received a second dose of TBPM-PI-HBr or placebo following a standardized meal. A second cohort of subjects (cohort 7) also received this dose and formulation in the fasted and fed state to ensure adequate PK data for analysis (Table 8).

**TABLE 8:** Doses and tebipenem formulation for each cohort

Phase	Cohort	No. (active:placebo)	TBPM-PI-HBr dose/formulation^*a*^
SAD	1	8 (6:2)	300 mg, 12-h ER, fasted/fed
	2	8 (6:2)	600 mg, 12-h ER, fasted/fed^*b*^
	3	8 (6:2)	900 mg, 12-h ER, fasted
	6	8 (6:2)	100 mg, 12-h ER, fasted
	7	8 (6:2)	600 mg, 12-h ER fasted/fed^*b*^
	8	8 (6:2)	300 mg, IR, fasted/fed
	9	8 (6:2)	300 mg, 2-h ER, fasted/fed
	10	8 (6:2)	300 mg, 4-h ER, fasted/fed
	11	8 (6:2)	300 mg, 6-h ER, fasted/fed
	12	8 (8:0)	300 mg (Orapenem), fasted/fed^*c*^
	13	8 (6:2)	600 mg, IR, fasted/fed
	14	8 (6:2)	600 mg, 4-h ER, fasted/fed
	15	8 (6:2)	Not used
	16	8 (6:2)	100 mg, IR, fasted
	17	8 (6:2)	900 mg, IR, fasted
MAD	4	8 (6:2)	300 mg, IR q8h for 14 days
	5	8 (6:2)	600 mg, IR q8h for 14 days

a TBPM-PI-HBr dosages indicate amounts of TBPM-PI; each 300-mg dose of TBPM-PI contains 231 mg of active TBPM.

b Initially, cohort 2 and cohort 7 were designed as receiving the same dosage of SPR994 (600-mg 12-h ER) under fasting-only and fasting/fed conditions, respectively. A protocol amendment allowed subjects in cohort 2 to return to the unit for repeated dosing in fed condition after a 5-day washout, in order to maximize the number of subjects with fed-condition dosing at this dose. Five of the 8 subjects in cohort 2 returned for this fed-condition dosing. These cohorts were combined for the fasted/fed PK analysis of the 600-mg 12-h ER dosing group.

c Orapenem dosage refers to 300 mg of active TBPM, or 389.1 mg of TBPM-PI granules. The Orapenem granule cohort was open label, not placebo controlled.

To compare the PK of TBPM-PI-HBr to those of the commercial preparation of TBPM-PI pediatric fine granules (Orapenem), which is approved for the treatment of respiratory infections in Japan, an additional open-label control cohort was included in which all 8 subjects received a single 300-mg oral dose of Orapenem (equivalent to 389.1 mg of TBPM-PI granules) following a 10-h fast and a second dose on day 7 in the fed state following a minimum 5-day washout period (Table 8).

Blinded safety data were reviewed by a safety monitoring group prior to each dose escalation. The decision to escalate to the next dose was governed by predefined criteria.

The MAD cohorts were enrolled following confirmation that the 300- and 600-mg dose levels were safe and well tolerated in SAD and that these doses produced plasma concentration-time profiles likely to be clinically effective based on preclinical pharmacodynamic assessments and clinical experience with Orapenem. In preclinical studies, *f*AUC/MIC·1/tau had the strongest predictive correlation with efficacy (15). IR tablets achieved the optimal balance of drug release rate versus limited human absorption window and total exposure requirements compared to various timed-release tablets (2, 4, 6, and 12 h). The longer-release profiles failed to provide adequate exposure, and shorter-release profiles provided no advantage over an IR formulation. Therefore, the IR tablet was selected for further evaluation in the MAD portion of the study. In the MAD phase, subjects received multiple doses of TBPM-PI-HBr in dosages of 300 mg or 600 mg every 8 h (q8h) or placebo for 14 consecutive days. Subjects fasted 2 h prior and 1 h after each dose administration in MAD.

### Subject selection.

Healthy adult subjects aged 18 to 55 years with a body mass index of 18.5 to 29.9 kg/m^2^ and weight between 55 and 100 kg were eligible. Subjects were medically healthy with no clinically significant abnormalities based on physical examination, vital signs (temperature, heart rate, blood pressure, and respiratory rate), ECG, and clinical laboratory testing (serum chemistry, hematology, and urinalysis). All subjects were nonsmokers, females were of nonchildbearing potential, and males used an acceptable form of contraception. Subjects were excluded for any clinically significant medical condition, a history of Clostridium difficile infection, positive test for human immunodeficiency virus (HIV), hepatitis B virus surface antigen (HBsAg), or hepatitis C antibodies (anti-HCV), positive urine drug/alcohol test or history of substance or alcohol abuse, documented hypersensitivity or anaphylaxis to any medication, or use of any prescription or over-the-counter medications with 7 days of randomization. In addition, subjects were required to have a QT wave corrected for heart rate (HR) using Fridericia’s method (QTcF) interval duration <450 ms, which was determined as an average from triplicate ECGs obtained at screening and predose day 1 after at least 5 min in a semisupine quiet resting state.

### Study assessments.

Safety assessments included adverse events, clinical laboratory testing (hematology, serum chemistry, and urinalysis), vital signs (blood pressure, heart rate, body temperature, and respiratory rate), physical examination, and triplicate 12-lead ECGs. In the SAD phase, estimated creatinine clearance (CL_CR_) was calculated at screening and on day −1 using the Cockcroft Gault equation. In the MAD phase, 24-h CL_CR_ based on plasma and urine creatinine concentration was determined prior to dosing and following the last dose on day 14. Serum creatinine concentrations were measured from the clinical laboratory tests performed on days −1 and 15. Urine creatinine concentration was measured using 24-h collections prior to the first dose on day 1 and over 24 to 48 h following the start of the last dose (day 14).

### Pharmacokinetic analysis.

Maximum plasma concentration (*C*_max_), area under the concentration-time curve from time zero to last measurable time point (AUC_0–_*_t_*), area under the concentration-time curve from time zero to infinity (AUC_0–∞_), time to maximum concentration (*T*_max_), terminal elimination rate constant (*k*_el_), terminal half-life (*t*_1/2_), terminal clearance (CL/F), volume of distribution (*V*/F), and *V* at steady-state (Vss/F) were determined for the SAD and MAD phases. In addition, area under the concentration-time curve from 0 to 8 h after the start of first dose (AUC_0–8_) was determined for the SAD phase and on day 1 for the MAD phase using noncompartmental methods. All pharmacokinetic calculations were performed using Phoenix WinNonlin version 8.0.

For the SAD phase, blood samples for determination of plasma concentrations of tebipenem were collected for fasted dose administration on day 1 and fed dose administration on day 7 predose and 0.25, 0.5, 0.75, 1, 1.5 (from cohort 8 onwards), 2, 4, 6, 8, 12, 24, and 48 h postdose. For the MAD phase, blood samples for tebipenem were collected on day 1 predose and at 0.25, 0.5, 0.75, 1, 1.5, 2, 4, 6, and 8 (just prior to second dose) hours postdose, predose on days 2, 3, 5, 7, 9, 11, and 13, and, for the last dose on day 14, predose and then at 0.25, 0.5, 0.75, 1, 1.5, 2, 4, 6, 8, 12, 24, 36, and 48 h postdose. The amount of total drug excreted in the urine was measured predose and at 0- to 4-h, 4- to 8-h, 8- to 12-h, and 12- to 24-h intervals after first dose on days 1 to 2 and 7 to 8 (food effect cohorts) in the SAD phase; urine concentrations were assumed to be unbound from protein. For the MAD phase, urine samples were collected predose, on day 1 at 0 to 4 h and 4 to 8 h prior to the second dose (q8h dosing), and on days 14 to 15 at 0 to 4 h, 4 to 8 h, and 12 to 24 h after the start of the last dose. Whole-blood and urine samples were assayed for total tebipenem concentrations using a validated liquid chromatography-tandem mass spectrometry (LC-MS/MS) method. The analytical range of the assay was 2.0 to 1,000 ng/ml in whole blood, and standards were fit to a weighted linear or power regression; stable labeled tebipenem pivoxil was used as an internal standard. Quality control concentrations included 2.00 ng/ml (lower limit of quantification [LLOQ]), 6.00 ng/ml (low), 40.0 ng/ml (middle), and 800 ng/ml (high); concentrations of tebipenem that were less than the LLOQ of the assay (2.00 ng/ml) were assigned a value of 0. Intrabatch accuracy and precision for the tebipenem validated assay were −7.0 to 5.1% bias and 2.5 to 7.4% coefficient of variation (CV), respectively; interbatch accuracy and precision were −4.0 to 1.0% bias and 4.4 to 6.2% CV, respectively. Dilution linearity was 5,000 ng/ml, with a dilution factor of 10.

### Statistical analysis.

The safety analysis population included all subjects who received study drug.

The PK population included all subjects with evaluable concentration-time profiles for each active dose who had no major protocol violations that impacted PK. Plasma concentrations and PK parameters for tebipenem were summarized for each treatment using descriptive statistics. Dose proportionality was assessed with linear models using fasted data from the SAD cohorts. Dose proportionality of log transformed *C*_max_ and AUC across the dose range was assessed by fitting the model log *C*_max_ (or AUC) = α + β × dose and testing for β = 1. This analysis was undertaken using the nominal dose administered. The effect of food on bioavailability was assessed using cohorts 1, 2, and 7. The PK of fed versus fasted dose administration in the same subjects was assessed by analysis of variance (ANOVA) of log-transformed *C*_max_, AUC_0–_*_t_*, and AUC_0–∞_ using a model with factors for treatment (fed status) and subject, separately by dose. Treatment mean differences and 90% confidence intervals (CI) of the log-transformed PK parameters were back-transformed to present the geometric ratio of least-squares means and 90% confidence limits. All statistical analyses were performed using SAS version 9.3.

## References

[1] CDC. Antibiotic resistance threats in the United States, 2013. https://www.cdc.gov/drugresistance/pdf/ar-threats-2013-508.pdf. Accessed 24 May 2016.

[2] WHO. Antibiotic resistance. http://www.who.int/mediacentre/factsheets/antibiotic-resistance/en/.

[3] Kadri SS, Adjemian J, Lai YL, Spaulding AB, Ricotta E, Prevots DR, Palmore TN, Rhee C, Klompas M, Dekker JP, Powers JH III, Suffredini AF, Hooper DC, Fridkin S, Danner RL, National Institutes of Health Antimicrobial Resistance Outcomes Research Initiative (NIH—ARORI). 2018 Difficult-to-treat resistance in Gram-negative bacteremia at 173 US hospitals: retrospective cohort analysis of prevalence, predictors, and outcome of resistance to all first-line agents. Clin Infect Dis 67:1803–1814. doi:10.1093/cid/ciy378. 30052813 PMC6260171

[4] Logan LK, Weinstein RA 2017 The epidemiology of carbapenem-resistant Enterobacteriaceae: the impact and evolution of a global menace. J Infect Dis 215(Suppl 1):S28–S36. doi:10.1093/infdis/jiw282. 28375512 PMC5853342

[5] Potter RF, D’Souza AW, Dantas G 2016 The rapid spread of carbapenem-resistant Enterobacteriaceae. Drug Resist Updat 29:30–46. doi:10.1016/j.drup.2016.09.002. 27912842 PMC5140036

[6] Citron DM, Tyrrell KL, Rubio A, Goldstein EJC 2018 *In vitro* activity of tebipenem (SPR859), tebipenem-pivoxil (SPR994) and meropenem against a broad spectrum of anaerobic bacteria, poster 559. Microbe 2018, American Society for Microbiology.

[7] Lacasse E, Brouillette E, Larose A, Parr TR, Rubio A, Malouin F 27 3 2019 *In vitro* activity of tebipenem (SPR859) against penicillin-binding proteins of gram-negative bacteria. Antimicrob Agents Chemother 63:e02181-18. doi:10.1128/AAC.02181-18. 30718255 PMC6437484

[8] Mendes RE, Rhomberg PR, Huynh H, Cotroneo N, Rubio A, Flamm RK 1 4 2019 Antimicrobial activity of tebipenem (SPR859) against a global challenge set. Antimicrob Agents Chemother 63:e02618-18. doi:10.1128/AAC.02618-18. 30936096 PMC6535533

[9] Mendes RE, Rhomberg PR, Watters A, Cotroneo N, Rubio A, Flamm RK 2018 Antimicrobial activity assessment of tebipenem (SPR859) against an isolate collection causing urinary tract infections, poster 558. Microbe 2018, American Society for Microbiology.

[10] Zou Y, Cotroneo N, Rubio A 2018 *In vitro* bactericidal activity and post-antibiotic effect of tebipenem (SPR859) against susceptible and extended-spectrum beta-lactamase producing Enterobacteriaceae as compared to levofloxacin (LVX) and meropenem (MEM), poster 561. Microbe 2018, American Society for Microbiology.

[11] Grosser L, Heang K, Teague J, Jain A, Warn P, Corbett D, Rubio A 2018 *In vivo* efficacy of tebipenem-pivoxil (SPR994) in an acute murine thigh infection caused by *Escherichia coli* and *Klebsiella pneumoniae*, poster 566. Microbe 2018, American Society for Microbiology.

[12] Heang K, Grosser L, Farrington K, Controneo N, Jain A, Cacarro L, Corbett D, Rubio A 2018 *In vivo* characterization of tebipenem-pivoxil (SPR994) in a murine ascending *Escherichia coli* urinary tract infection model, poster 565. Microbe 2018, American Society for Microbiology.

[13] Teague J, Corbet D, Burgess E, Williams J, Evenden P, Daws G, Vaccaro L, Jain A, Warn P, Rubio A 2018 *In vivo* efficacy of tebipenem-pivoxil (SPR994) in neutropenic murine lung models of gram-negative bacterial infection, poster 567. Microbe 2018, American Society for Microbiology.

[14] Johnson A, Farrington N, McEntee L, Kirby A, Melnick D, Rubio A, Utley L, Parr T, Hope W, Das S 2018 Pharmacokinetics and pharmacodynamics of tebipenem (SPR859) for multi-drug resistant Enterobacteriaceae in a hollow fibre infection model, poster L2. ASM/ESCMID 2018, American Society for Microbiology.

[15] McEntee L, Farrington N, Johnson A, Gore S, Rubio A, Utley L, Parr T, Ambrose P, Das S, Hope W 2018 Pharmacokinetics and pharmacodynamics of tebipenem for multi-drug resistant Enterobacteriaceae, poster L3. ASM/ESCMID 2018, American Society for Microbiology.

